# An RNA Recognition Motif-Containing Protein Functions in Meiotic Silencing by Unpaired DNA

**DOI:** 10.1534/g3.117.041848

**Published:** 2017-06-30

**Authors:** Dilini A. Samarajeewa, Pennapa Manitchotpisit, Miranda Henderson, Hua Xiao, David G. Rehard, Kevin A. Edwards, Patrick K. T. Shiu, Thomas M. Hammond

**Affiliations:** * School of Biological Sciences, Illinois State University, Normal, Illinois 61790; † Division of Biological Sciences, University of Missouri, Columbia, Missouri 65211

**Keywords:** meiosis, chromosome pairing, RNA silencing, homology search, RRM domain

## Abstract

Meiotic silencing by unpaired DNA (MSUD) is a biological process that searches pairs of homologous chromosomes (homologs) for segments of DNA that are unpaired. Genes found within unpaired segments are silenced for the duration of meiosis. In this report, we describe the identification and characterization of *Neurospora crassa sad-7*, a gene that encodes a protein with an RNA recognition motif (RRM). Orthologs of *sad-7* are found in a wide range of ascomycete fungi. In *N. crassa*, *sad-7* is required for a fully efficient MSUD response to unpaired genes. Additionally, at least one parent must have a functional *sad-7* allele for a cross to produce ascospores. Although *sad-7*-null crosses are barren, *sad-7*^Δ^ strains grow at a wild-type (wt) rate and appear normal under vegetative growth conditions. With respect to expression, *sad-7* is transcribed at baseline levels in early vegetative cultures, at slightly higher levels in mating-competent cultures, and is at its highest level during mating. These findings suggest that SAD-7 is specific to mating-competent and sexual cultures. Although the role of SAD-7 in MSUD remains elusive, green fluorescent protein (GFP)-based tagging studies place SAD-7 within nuclei, perinuclear regions, and cytoplasmic foci of meiotic cells. This localization pattern is unique among known MSUD proteins and raises the possibility that SAD-7 coordinates nuclear, perinuclear, and cytoplasmic aspects of MSUD.

During meiosis in eukaryotic organisms, homologous chromosomes are grouped into pairs, aligned, recombined, and segregated to produce genetically variable nuclei for reproduction. Chromosome alignment during this process may provide meiotic cells with an opportunity to identify potential problems within pairs of homologs. For example, consider a transposon that exists between two genes on one chromosome but not between the same two genes on the chromosome’s homolog. Alignment of the homologs would pair the genes flanking the transposon, while the transposon itself would remain unpaired. A few fungi have been shown to possess the ability to detect unpaired DNA, like the aforementioned transposon, and silence it for the duration of meiosis. In these fungi, the process is called MSUD (Aramayo and Metzenberg 1996; Shiu *et al.* 2001; Son *et al.* 2011; Nagasowjanya *et al.* 2013; Wang *et al.* 2015).

MSUD was initially discovered in *Neurospora crassa* (Aramayo and Metzenberg 1996; Shiu *et al.* 2001), a filamentous fungus made famous as a research model by Beadle and Tatum (1941). Although *N. crassa* is haploid for most of its life cycle, it possesses a brief diploid phase that occurs during sexual reproduction. MSUD begins during this diploid phase and continues throughout meiosis; thus, a brief introduction to *N. crassa*’s sexual cycle is necessary [please see Raju (1980) for a comprehensive review of the *N. crassa* sexual cycle].

In *N. crassa*, the sexual cycle begins with the formation of an immature fruiting body called a protoperithecium. A hair-like cell structure called a trichogyne then extends from the protoperithecium toward an asexual spore (conidium) or a hyphal segment of a strain of the opposite mating type. Fertilization begins when fusion occurs and a “male” nucleus travels through the trichogyne to the protoperithecium. The protoperithecium becomes a perithecium after fertilization. Within the perithecium, the parental nuclei replicate and, through a series of coordinated events, a nucleus from each parent is sequestered at the top of a cell structure called a crozier. The two haploid parental nuclei fuse to form a single diploid nucleus while the tip of the crozier elongates to form a tube-like meiotic cell. After nuclear fusion, the seven chromosomes from each parent are paired, aligned, and recombined. Segregation during meiosis I returns the haploid state and meiosis II produces four meiotic products. These meiotic products then undergo a single round of mitosis to produce a total of eight nuclei in a single meiotic cell. Cell walls and membranes develop around each of the eight nuclei during a process called ascosporogenesis. At this stage, the meiotic cell is generally referred to as an ascus (“spore sac”). A perithecium can have hundreds of asci, each formed from a unique meiotic event. At maturity, ascospores are shot from the perithecium. This results in the accumulation of ascospores on the underside of a crossing lid when mating is conducted in a standard petri dish.

The path to MSUD discovery began with the deletion of a gene called *ascospore maturation-1* (*asm-1*) (Aramayo and Metzenberg 1996; Hammond 2017). The *asm-1* gene is required for proper ascospore maturation and its loss results in the production of white ascospores (Aramayo *et al.* 1996). Interestingly, even in *asm-1*^+^ × *asm-1*^Δ^ crosses, where four of eight ascospores in each ascus inherit an *asm-1*^+^ allele, asci typically contain eight white ascospores instead of four black and four white ascospores (Aramayo and Metzenberg 1996). This phenotype occurs because MSUD detects *asm-1*^+^ as unpaired and silences it throughout meiosis (Shiu *et al.* 2001; Shiu and Metzenberg 2002).

Genes like *asm-1* are often targeted in MSUD experiments because they allow for MSUD efficiency to be quantified with ascospore phenotype. For example, if a mutation that suppresses MSUD is included in an *asm-1*^+^ × *asm-1*^Δ^ cross, the strength of MSUD suppression can be determined by the percentage of black ascospores produced by the cross (Lee *et al.* 2003, 2010; Xiao *et al.* 2010; Hammond *et al.* 2011a, 2013b; Samarajeewa *et al.* 2014). A strong MSUD suppressor will produce a high percentage of black ascospores (because the unpaired *asm-1*^+^ allele is expressed), while a weak MSUD suppressor will produce a low percentage of black ascospores (because the unpaired *asm-1*^+^ allele is mostly silenced). In addition to *asm-1*, a gene called *Round spore* (*r*) is often utilized in MSUD research because it must be expressed during meiosis for a cross to produce spindle-shaped ascospores. Accordingly, MSUD causes *r*^+^ × *r*^Δ^ crosses to produce round ascospores (Shiu *et al.* 2001; Pratt *et al.* 2004). The level of MSUD suppression can be quantified in *r*^+^ × *r*^Δ^ crosses similar to the way it is quantified in *asm-1*^+^ × *asm-1*^Δ^ crosses (Xiao *et al.* 2010; Hammond *et al.* 2011a, 2013b; Samarajeewa *et al.* 2014). In an *r*^+^ × *r*^Δ^ crossing background, a strong MSUD suppressor will produce a high percentage of spindle ascospores (because *r*^+^ is expressed) and a weak MSUD suppressor will produce a low percentage of spindle ascospores (because *r*^+^ is mostly silenced).

In *asm-1*^+^ × *asm-1*^Δ^ and *r*^+^ × *r*^Δ^ crosses, the corresponding unpaired regions are only 2.3 and 3.5 kb, respectively (Colot *et al.* 2006). Furthermore, there is evidence suggesting that MSUD can identify unpaired DNA segments as short as 1.3 kb (Lee *et al.* 2004). Efforts to determine how MSUD detects such relatively small segments of unpaired DNA have focused on identifying silencing factors through genetic screens. These efforts have so far identified eleven silencing proteins. A model for the MSUD mechanism has been developed from cytological analyses of these fluorescently-tagged proteins and inferences based on the functions of their homologs in other systems (Aramayo and Selker 2013; Hammond 2017).

MSUD begins with the identification of unpaired DNA through an undetermined mechanism, possibly involving nuclear MSUD proteins SAD-5 and SAD-6 (Hammond *et al.* 2013b; Samarajeewa *et al.* 2014). While SAD-5 lacks characterized domains and homologs with described functions, SAD-6 contains an SNF2 helicase domain and is related to proteins that mediate DNA homology search processes. After an unpaired DNA is detected, hypothetical molecules called aberrant RNAs (aRNAs) are thought to be transcribed from the unpaired region and delivered to perinuclear MSUD proteins docked along the nuclear envelope (Bardiya *et al.* 2008; Decker *et al.* 2015). These molecules are called aRNAs because they are assumed to be unique or marked in a manner that allows the cell to distinguish them from “normal” RNAs. The perinuclear MSUD proteins include SAD-1, an RNA-directed RNA polymerase (Shiu *et al.* 2001, 2006); DCL-1, a Dicer homolog (Alexander *et al.* 2008); QIP, an exonuclease (Maiti *et al.* 2007; Lee *et al.* 2010; Xiao *et al.* 2010); and SMS-2, an Argonaute protein (Lee *et al.* 2003). Presumably, SAD-1 synthesizes double-stranded (ds)RNAs from aRNAs, DCL-1 dices dsRNAs into MSUD-associated small interfering RNAs (masiRNAs) (Hammond *et al.* 2013a), QIP processes masiRNAs into single strands, and SMS-2 uses the single-stranded masiRNAs as guides to identify complementary RNA molecules for silencing. Other perinuclear MSUD proteins include SAD-2, which is required for recruiting most if not all known perinuclear MSUD proteins to the perinuclear region of the meiotic cell (Shiu *et al.* 2006; Decker *et al.* 2015); SAD-3, which has a helicase-like domain and is a homolog of a protein involved in RNAi-mediated heterochromatin formation in *Schizosaccharomyces pombe* (Hammond *et al.* 2011a); and SAD-4, a novel protein required for masiRNA production (Hammond *et al.* 2013a). The most recent additions to the MSUD model are the nuclear cap-binding proteins CBP20 and CBP80, which may help deliver capped RNA molecules to the perinuclear silencing machinery (Decker *et al.* 2017).

Although the hypothetical aRNAs and dsRNAs of MSUD have not been detected biochemically, masiRNAs have been identified by RNA sequencing (Hammond *et al.* 2013a; Wang *et al.* 2015). These molecules are predominantly 25 nucleotides long with a bias for uridine at their 5′ ends. It seems likely that masiRNAs are used to silence any complementary RNA molecules, not just those derived from the unpaired DNA. This would explain why unpaired genes trigger silencing of paired copies of the same genes at other locations in the genome (Shiu *et al.* 2001; Lee *et al.* 2004).

While the working model of MSUD is consistent with current observations, it leaves many questions unanswered. For example, how are homologous chromosomes scanned and how is unpaired DNA identified? If aRNAs exist, how are they transferred from unpaired DNA to the perinuclear region? How do perinuclear MSUD proteins distinguish aRNAs from mRNAs? Answering these and the many other outstanding questions on MSUD will likely require the discovery of additional MSUD proteins through genetic screens for silencing suppressors.

Previously, Hammond *et al.* (2011a) described a high-throughput reverse genetic screen to identify suppressors of MSUD. The screen involves crossing strains from the *N. crassa* knockout library (Colot *et al.* 2006) with strains that have been genetically engineered to unpair *asm-1* or *r* during meiosis. A strain from the knockout library is marked as a candidate MSUD suppressor if it increases the production of black ascospores (when *asm-1* is unpaired) or spindle ascospores (when *r* is unpaired). A suppressor candidate is then examined with a series of experiments designed to purify the knockout strain from possible contaminants and confirm that the gene deletion associated with the knockout strain is responsible for the MSUD suppression phenotype. The gene is then characterized to help understand why its loss suppresses MSUD. Here, we report using the screen to identify *sad-7*, a gene required for a fully efficient MSUD response to unpaired DNA.

## Materials and Methods

### Strains, media, culture conditions, crosses, and general techniques

The key strains used in this study are listed along with genotype information in Table 1. The *N. crassa* knockout collection (Colot *et al.* 2006) and various markers were obtained from the Fungal Genetics Stock Center (FGSC) (McCluskey *et al.* 2010). Strains were cultured on Vogel’s medium (Vogel 1956), except when performing a cross. Crosses were conducted on synthetic crossing medium (pH 6.5; Westergaard and Mitchell 1947) with 1.5% sucrose. Experiments and sexual crosses were performed on a laboratory bench top at room temperature with ambient lighting unless otherwise indicated. Genomic DNA was isolated from lyophilized mycelia using IBI Scientific’s Mini Genomic DNA Kit (Plant/Fungi). When necessary, PCR products were purified with IBI Scientific’s Gel/PCR DNA Fragment Extraction Kit. PCR was generally performed with Thermo Scientific’s Phusion High Fidelity DNA Polymerase.

**Table 1:** Strains used in this study

Strain Name	Genotype
F2-26 (RTH1005.2)	*rid*; *fl a*
F2-27 (RTH1027.3)	*rid r*^∆^::*hph*; *fl a*
F3-24 (RTH1083.17)	*rid his-3*^+^::*asm-1*; *fl*; *asm-1*^Δ^::*hph a*
FGSC 13880	*sad-7*^∆^::*hph a*
ISU-3329 (RDS19.3)	*rid*; *fl*; *mus-52*^∆^::*bar mCherryNC-spo76*::*hph*; *sad-2*^∆^::*hph A*
ISU-3334 (RDS19.9)	*rid*; *fl*; *mus-52*^∆^::*bar mCherryNC-spo76*::*hph*; *sad-2*^∆^::*hph a*
ISU-3817 (HDS30.1.1)	*rid gfp-sad-7*::*hph his-3*; *mus-52*^∆^::*bar A*
ISU-4078 (HDS34.1.2)	*rid gfp-sad-7*^∆1-67^::*hph his-3*; *mus-52*^∆^::*bar A*
ISU-4079 (HDS35.1.1)	*rid gfp-sad-7*^∆1-118^::*hph his-3*; *mus-52*^∆^::*bar A*
ISU-4134 (RAB1.8)	*rid A*
ISU-4217 (HDS36.1.1)	*rid gfp-sad-7*^∆1-206^::*hph his-3*; *mus-52*^∆^::*bar A*
ISU-4261 (RTH0061.2.1.6)	*rid his-3 gfp-sad-3*::*hph*; *mus-52*^∆^::*bar?*; *mus-51*^∆^::*bar? a*
ISU-4262 (RTH1035.7)	*rid sad-7*^∆^::*hph A*
ISU-4263 (P16-17)	*a*
ISU-4264 (F5-23)	*fl A*
ISU-4265 (RTH1080.19)	*sad-7*^∆^::*hph*; *fl a*
P6-07	*rid A*
P6-08	*rid a*
P8-01	*sad-2*^Δ^::*hph A*
P8-42	*rid his-3*; *mus-51*^∆^::*bar a*
P8-43	*rid his-3*; *mus-52*^∆^::*bar A*
P15-22	*rid his-3*; *mus-52*^∆^::*bar*; *gfp-sms-2*::*hph A*

All strains in this study are descendants of lines 74-OR23-1VA (FGSC 2489) and 74-ORS-6a (FGSC 4200) (Perkins 2004). The *mCherryNC-spo76*::*hph* allele was obtained from ISU-3123 (Samarajeewa *et al.* 2014). ISU-4261 carries a *gfp-sad3*::*hph* allele identical to the one contained in strain F4-31 (Hammond *et al.* 2011a). The *gfp-sms2*::*hph* allele in P15-22 was described in Hammond *et al.* (2011b). The *r*^∆^, *asm-1*^∆^, *sad-7*^∆^, *mus-51*^∆^, and *mus-52*^∆^ alleles are as described by Colot *et al.* (2006). Mutant *rid* alleles suppress repeat-induced point mutation (Freitag *et al.* 2002). The *fl* allele eliminates macroconidia production (Perkins *et al.* 2000).

### Genetic modification of N. crassa

Transformation of conidia was performed by electroporation with the method of Margolin *et al.* (1997). Conidia were filtered through a 100 μm nylon filter (EMD Millipore, SCNY00100) before collection by centrifugation. Strain P8-43 was used as the transformation host to attach the gene for green fluorescent protein (*gfp*) to *sad-7* at its endogenous location on chromosome I, as previously described (Hammond *et al.* 2011b). Four different *gfp-sad-7* fusions were constructed. The *gfp* coding region was fused to either the first, 68th, 119th, or 207th codon of *sad-7*. In each case, the GFP tag was placed on the N-terminus of SAD-7 (or truncated SAD-7). After transformation, the *gfp-sad-7* coding regions were PCR-amplified from the transgenes and determined to be free of mutations by Sanger sequencing (data not shown). Transgene vectors were constructed with double-joint PCRs (Yu *et al.* 2004; Hammond *et al.* 2011b) using primers described in Supplemental Material, Table S1 in File S1.

### Gene expression analysis

A total of 23 RNA sequencing datasets were downloaded from the Sequence Read Archive of the National Center for Biotechnology Information (NCBI) (Leinonen *et al.* 2011). Ellison *et al.* (2011) generated datasets 1–3 from poly-A RNA; Wu *et al.* (2014) generated datasets 4–13 from poly-A RNA; Wang *et al.* (2014) generated datasets 14–21 from poly-A RNA; and Samarajeewa *et al.* (2014) generated datasets 22 and 23 from rRNA-reduced total RNA. Although MSUD gene expression levels for some of the 23 datasets were previously examined by Samarajeewa *et al.* (2014), Wang *et al.* (2014), and Decker *et al.* (2017), *sad-7* expression levels were not examined in any of the three studies, so the datasets were reanalyzed and presented here. The culture methods used to generate each dataset are briefly described in the *Results* section. Complete culture methods can be obtained from the original reports. Reads from each dataset were aligned to all primary transcripts located in version 12 of the *N. crassa* genome annotation, which was provided by the Broad Institute of MIT and Harvard (Galagan *et al.* 2003). Read alignments were performed with Bowtie 2 v.2.2.5 (Langmead and Salzberg 2012) using the local alignment setting. RPKM (Reads per kilobase exon model per million mapped reads; Mortazavi *et al.* 2008) values were calculated from read alignments with custom Perl scripts. Reads aligning to more than one location and/or containing more than one mismatch were ignored. Accession numbers for the analyzed datasets are as follows: (1) SRR090363, (2) SRR090364, (3) SRR090366, (4) SRR1055985, (5) SRR1055990, (6) SRR1055986, (7) SRR1055991, (8) SRR1055987, (9) SRR1055992, (10) SRR1055988, (11) SRR1055993, (12) SRR1055989, (13) SRR1055994, (14) SRR585661, (15) SRR585662, (16) SRR585663, (17) SRR585664, (18) SRR585665, (19) SRR585666, (20) SRR585667, (21) SRR585668, (22) SRR957218, and (23) SRR957223.

### Confocal microscopy

Six-day-old perithecia were harvested from crossing plates and fixed in a solution of 4% paraformaldehyde, 100 mM PIPES at pH 6.9, 10 mM EGTA, and 5 mM MgSO_4_ at room temperature for 20 min before washing and storing in sodium phosphate buffer (80 mM Na_2_HPO_4_ and 20 mM NaH_2_PO_4_). Asci were dissected from perithecia in 25% glycerol and transferred by pipette to a drop of mounting medium (25% glycerol, 10 mg/ml DABCO, and 100 mM potassium phosphate buffer at pH 8.7) on a microscope slide. Cover slips were placed over the samples and sealed with clear nail polish after excess mounting medium was wicked away with tissue paper. Slides were stored at −20° before analysis with a Leica SP2 confocal microscope.

### Data availability

All strains generated during this study are available upon request. The authors state that all data necessary for confirming the conclusions presented in the article are represented fully within the article.

## Results

### Identification of an MSUD suppressor

**Figure 1:**
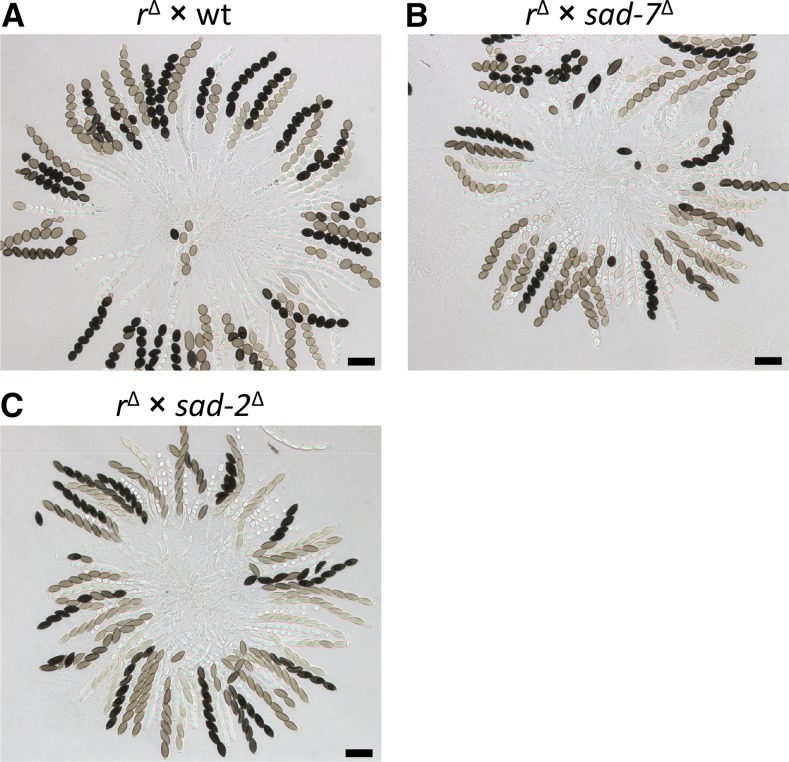
Meiotic silencing by unpaired DNA (MSUD) is suppressed in *sad-7*^Δ^ heterozygous crosses. (A) Asci from an *r*^Δ^ × wt (wild-type) perithecium. Mature (black-pigmented) ascospores are round because of MSUD. (B) Asci from an *r*^Δ^ × *sad-7*^Δ^ perithecium. Some mature ascospores are spindle-shaped because MSUD is partially suppressed by *sad-7*^Δ^. (C) Asci from an *r*^Δ^ × *sad-2*^Δ^ perithecium. Mature ascospores are predominantly spindle-shaped because *sad-2*^Δ^ is a strong suppressor of MSUD. The designated female strain in all crosses (*r*^Δ^ ♀) is F2-27. The designated male strains are wt ♂ P6-07, *sad-7*^Δ^ ♂ ISU-4262, and *sad-2*^Δ^ ♂ P8-01. Bars, 50 µm. Quantitative analysis of ascospore phenotypes from each cross is provided in Table 2.

Strain FGSC 13880 from the *N. crassa* knockout library was marked as a putative MSUD suppressor during a screen of mutants in the *N. crassa* knockout collection. FGSC 13880 is an *ncu01917* deletion mutant (*ncu01917*^Δ^), where *ncu01917* refers to a gene encoding a hypothetical protein on chromosome I. To confirm that loss of *ncu01917* from one parent of a cross suppresses MSUD, we performed quantitative MSUD suppression assays by crossing a *sad-2*^Δ^ strain, an *ncu01917*^Δ^ strain (a descendant of FGSC 13880), and a wt control strain with an *r*^Δ^ tester. The *sad-2*^Δ^ allele is among the strongest known suppressors of MSUD (Shiu *et al.* 2006). We found that *sad-2*^Δ^, *ncu01917*^Δ^, and wt produced 96.1, 53.2, and 1.7% spindle ascospores, respectively (Figure 1 and Table 2), thus silencing of *r*^+^ in a cross was inefficient when *sad-2*^Δ^ was a parent (most ascospores were spindles), more efficient when *ncu01917*^Δ^ was a parent (approximately half of the ascospores were spindles), and most efficient when wt was a parent (few ascospores were spindles). These results suggest that *ncu01917*^Δ^ suppresses silencing of unpaired *r*^+^, albeit not as strongly as *sad-2*^Δ^ does. Next, we repeated the crosses with an *asm-1*^Δ^ tester. We found that *sad-2*^Δ^, *ncu01917*^Δ^, and wt produced 60.2, 67.3, and 5.9% black ascospores in these crosses, respectively (Table 2); thus, silencing of unpaired *asm-1*^+^ was inefficient with *sad-2*^Δ^, similarly inefficient with *ncu01917*^Δ^, and highly efficient with wt. These results suggest that *ncu01917*^Δ^ suppresses silencing of *asm-1*^+^ as well as *sad-2*^Δ^ does, because approximately equal percentages of black ascospores were produced in *ncu01917*^Δ^ × *asm-1*^Δ^ and *sad-2*^Δ^ × *asm-1*^Δ^ crosses. A hypothesis to explain why *ncu01917*^Δ^ and *sad-2*^Δ^ suppress MSUD equally with respect to unpaired *asm-1*^+^ but not unpaired *r*^+^ is discussed below. Overall, these results suggest that *ncu01917*^Δ^ is a genuine suppressor of MSUD. To be consistent with the historical naming system for suppressors of MSUD (Shiu *et al.* 2001), we refer to *ncu01917* as *sad-7* (*suppressor of ascus dominance-7*) hereafter.

**Table 2:** MSUD is suppressed by *sad-7*^∆^

	wt ♀ Total (× 10^6^)	*r*^Δ^ ♀ Spindle (%)	*asm-1*^Δ^ ♀ Black (%)
wt ♂	8.3 ± 1.0	1.7 ± 1.1	5.9 ± 0.7
*sad-7*^∆^ ♂	8.9 ± 0.2	53.2 ± 6.5	67.3 ± 4.3
*sad-2*^∆^ ♂	8.7 ± 0.3	96.1 ± 1.1	60.2 ± 21.6

Unidirectional crosses were performed between MSUD-testers (females) and *wt*, *sad-7*^∆^, or *sad-2*^∆^ (males) as previously described (Samarajeewa *et al.* 2014). In short, crosses were performed in triplicate and ascospores were collected from the lids of crossing plates at 21 d postfertilization. Ascospores were suspended in water for analysis under magnification. The following phenotypes were analyzed: total ascospores (column 2), percent spindle ascospores (column 3), and percent black ascospores (column 4). Only the pertinent genotype is provided for each crossing parent. Note that “wt” is not a true wild-type but carries wild-type alleles for all genes related to MSUD, *r*, and *asm-1*. Strains: wt ♀ F2-26, *r*^∆^ ♀ F2-27, *asm-1*^∆^ ♀ F3-24, wt ♂ P6-07, *sad-7*^∆^ ♂ ISU-4262, and *sad-2*^∆^ ♂ P8-01. wt, wild-type; MSUD, meiotic silencing by unpaired DNA.

### SAD-7 is required for sexual development

**Figure 2:**
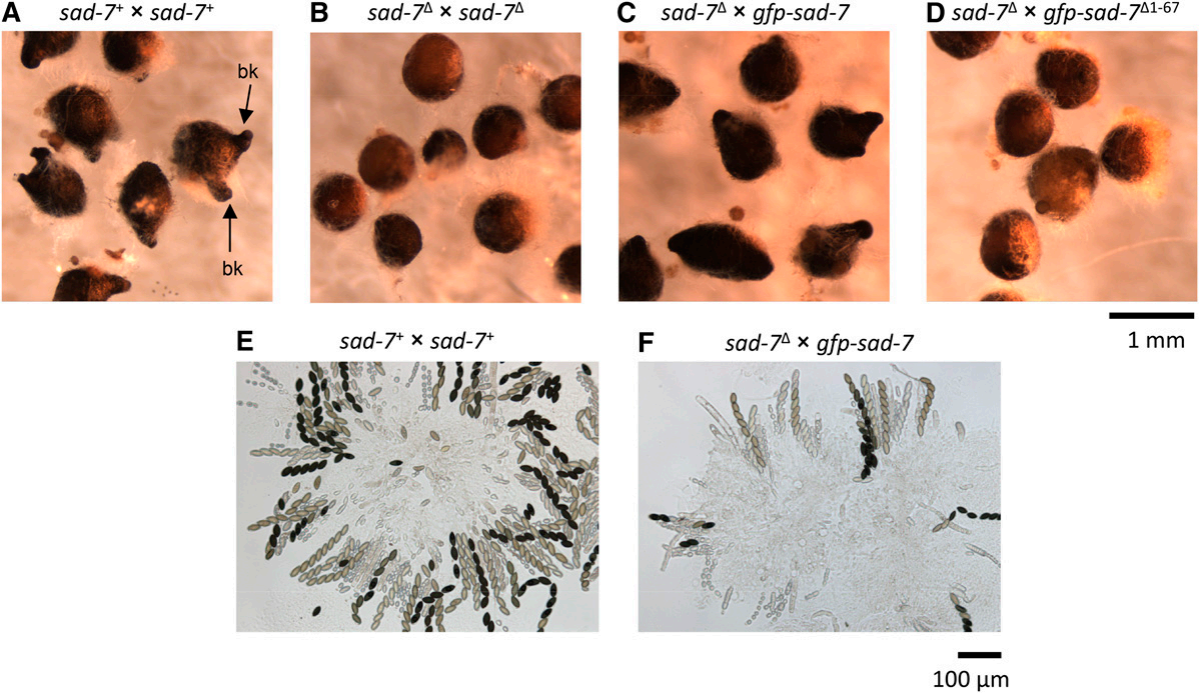
SAD-7 is required for ascus and ascospore development. (A–D) Perithecia were isolated from crosses 20 d postfertilization and examined in water under magnification. (A) Perithecia from a *sad-7*^+^ × *sad-7*^+^ cross (ISU-4264 × ISU-4263). Perithecial beaks (bk) are present, and two are highlighted in the image. (B) Perithecia from a *sad-7*^Δ^ × *sad-7*^Δ^ cross (ISU-4265 × ISU-4262). No beaks are observed. (C) Perithecia from a *sad-7*^Δ^ × *gfp-sad-7* cross (ISU-4265 × ISU-3817). Perithecial beak development appears normal. (D) Perithecia from a *sad-7*^Δ^ × *gfp-sad-7*^Δ1–67^ cross (ISU-4265 × ISU-4078). No beaks are observed. (E) Asci from a *sad-7*^+^ × *sad-7*^+^ cross (ISU-4264 × ISU-4263). Phenotypically normal asci and ascospores are detected. (F) Asci from a *sad-7*^Δ^ × *gfp-sad-7* cross (ISU-4265 × ISU-3817). Phenotypically normal asci and ascospores are detected. In summary, these results demonstrate that (1) at least one parent must have a functional SAD-7 protein for a cross to complete the sexual cycle and (2) tagging the full-length SAD-7 with GFP at its N-terminal end does not prevent the protein from performing its function in sexual reproduction.

The above findings demonstrate that heterozygous *sad-7*^Δ^ crosses are MSUD-deficient despite the presence of a *sad-7*^+^ allele in one parent. These findings are consistent with at least two hypotheses: (1) SAD-7 is critical for MSUD and decreased levels of the protein during meiosis interfere with MSUD function, and (2) SAD-7 is an auxiliary MSUD protein that improves the efficiency of MSUD but is not absolutely required for the process. In a *sad-7*^Δ^ × *sad-7*^Δ^ cross, the first hypothesis is supported if MSUD is completely inactive, while the second is supported if MSUD is partially active. We attempted to distinguish between these two hypotheses by first examining the ability of *sad-7*^Δ^ strains to complete the sexual cycle. In this experiment, *sad-7*^Δ^ × *sad-7*^Δ^ crosses were compared side-by-side with *sad-7*^+^ × *sad-7*^+^ crosses. We examined the perithecia of both at 20 d postfertilization and found that *sad-7*^+^ perithecia had normal beaks while *sad-7*^Δ^ perithecia were beakless (Figure 2, A and B). Upon dissection, we found that perithecia of *sad-7*^+^ × *sad-7*^+^ crosses contained hundreds of asci, most with mature or maturing ascospores (Figure 2E), while *sad-7*^Δ^ × *sad-7*^Δ^ perithecia were lacking asci and ascospores (data not shown; we encountered a few asci with immature ascospores in one rosette of a *sad-7*^Δ^ × *sad-7*^Δ^ cross, but we were unable to identify others during an examination of over 100 perithecia). These results are consistent with our inability to detect ascospores in a quantitative assay of sexual reproduction by *sad-7*^Δ^ × *sad-7*^Δ^ crosses (Table 3). Unfortunately, because at least one *sad-7*^+^ allele is required for completion of the sexual cycle, we were unable to determine if SAD-7 plays a critical or auxiliary role in MSUD.

**Table 3:** Homozygous *sad-7*^Δ^ crosses fail to produce ascospores

Cross	Total Ascospores (× 10^6^)
*sad-7*^Δ^ ♀ × wt ♂	3.6 ± 1.6
*sad-7*^Δ^ ♀ × *sad-7*^Δ^ ♂	0
*sad-7*^Δ^ ♀ × *sad-2*^Δ^ ♂	3.7 ± 0.1

Unidirectional crosses were performed between *sad-7*^Δ^ (female) and *wt*, *sad-7*^Δ^, or *sad-2*^Δ^ strains (males) as described in Table 2 to determine how *sad-7*^Δ^ affects ascospore production. Only the pertinent genotype is provided for each crossing parent. Please see Table 1 for complete genotype information. Strains: *sad-7*^Δ^ ♀ ISU-4265, wt ♂ P6-07, *sad-7*^Δ^ ♂ ISU-4262, and *sad-2*^Δ^ ♂ P8-01. wt, wild-type.

### SAD-7-null cultures are indistinguishable from wt cultures under standard growth conditions

**Figure 3:**
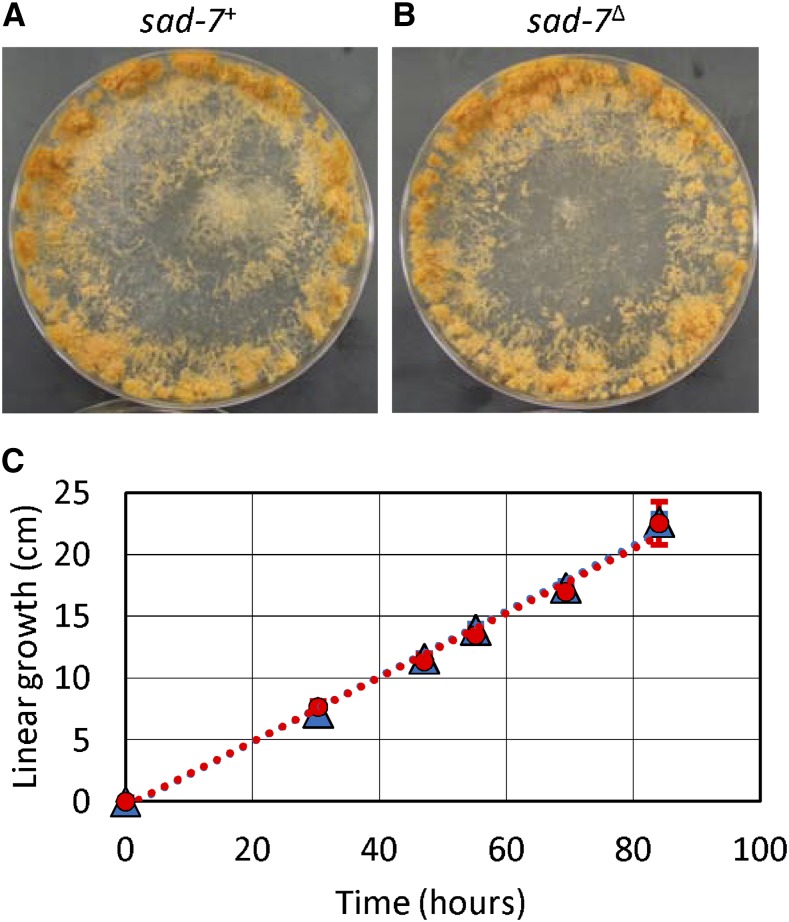
A *sad-7*^+^allele is not required for conidiogenesis or linear growth. (A and B) Cultures of *sad-7*^+^ (P6-08) and *sad-7*^Δ^ (ISU-4262) are indistinguishable when incubated on standard growth medium at room temperature on a laboratory bench top. (C) *sad-7*^+^ (ISU-4134, red circles) and *sad-7*^Δ^ (ISU-4262, blue triangles) have similar growth rates on standard growth medium at room temperature on a laboratory bench top. Linear growth rate was measured with a race tube assay (Perkins and Pollard 1986). Strains were allowed ∼2 d to colonize the race tubes before data collection. Error bars are SD values.

The discovery of SAD-7 brings the current number of known MSUD proteins to 12. Of the previous 11, none have been found to be required for normal growth or conidiogenesis under standard growth conditions. Thus, we examined if the loss of SAD-7 would affect growth or conidia production. First, when *sad-7*^+^ and *sad-7*^Δ^ strains were point-inoculated to the center of petri dishes containing standard medium and cultured for several days on a laboratory bench top, both grew at similar rates and produced qualitatively similar levels of conidia (Figure 3, A and B). Second, when linear growth rate was examined by inoculating *sad-7*^+^ and *sad-7*^Δ^ strains to the ends of 30 cm glass tubes containing standard growth medium (race tubes), both strains grew with the same maximum linear growth rate (Figure 3C). These data demonstrate that the deletion of *sad-7*^+^ does not alter growth rate or conidiogenesis (at least with respect to macroconidia) under standard growth conditions.

### The gene expression pattern of SAD-7 is similar to that of SAD-4

**Figure 4:**
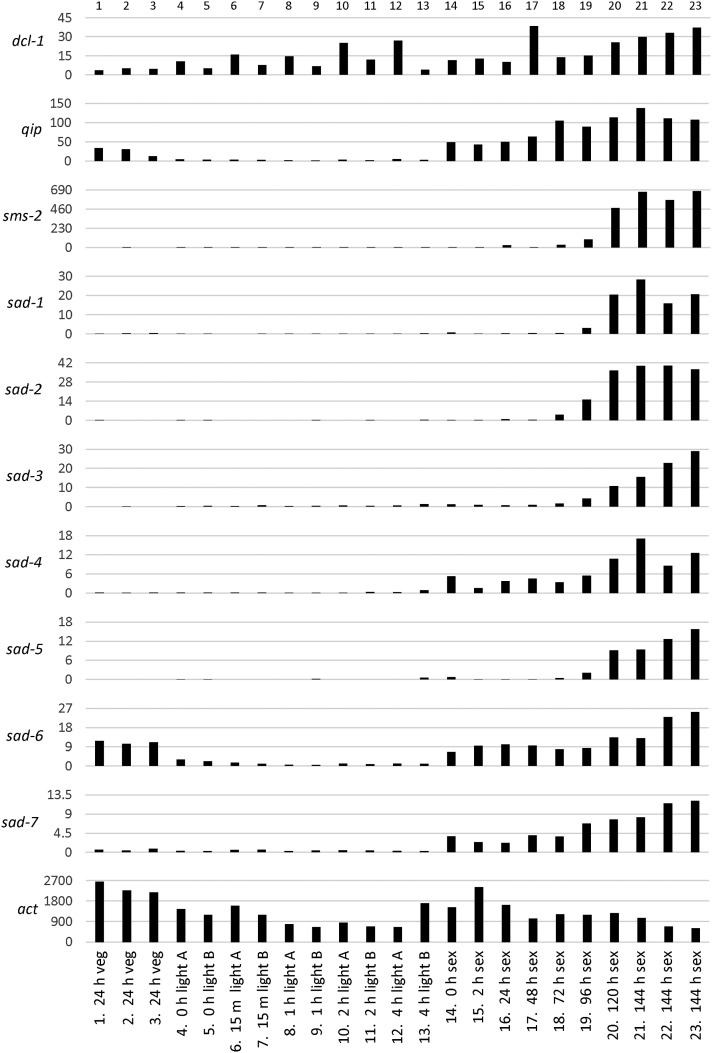
Expression pattern of *sad-7* is most similar to that of *sad-4*. The transcript levels of meiotic silencing by unpaired DNA (MSUD) genes under different culture conditions (according to RNA sequencing analysis) are shown. The *y*-axis marks the expression level of each gene in reads per kilobase exon model per million mapped reads (RPKM). The 23 datasets included in the analysis (Ellison *et al.* 2011; Samarajeewa *et al.* 2014; Wang *et al.* 2014; Wu *et al.* 2014) are plotted along the *x*-axis. See *Materials and Methods* and *Results* for a full description of each dataset. In short, datasets 1–3 are of vegetative cultures on solid medium, datasets 4–13 are of vegetative cultures in liquid medium, and datasets 14–23 are of sexual cultures on solid medium. For datasets 1–3, time refers to the age of the vegetative tissue; datasets 4–13, time refers to hours after exposure to light; and datasets 14–23, time refers to hours postfertilization. The “A” and “B” designations refer to replicate datasets that were generated with slightly different methods after RNA isolation (Wu *et al.* 2014). Five of the datasets (1–3, 22, and 23) used in this study were examined by Samarajeewa *et al.* (2014) and eight (14–21) were examined by Wang *et al.* (2014) with respect to MSUD gene expression, but *sad-7* expression was not examined in either study. Overall, *sad-7*’s expression pattern is most similar to that of *sad-4*, with barely detectable expression during early vegetative culture conditions (datasets 1–13), elevated expression in protoperithecial cultures (dataset 14), and maximum expression in sexual cultures (datasets 15–23). Gene numbers: *dcl-1* (*ncu08270*); *qip* (*ncu00076*); *sms-2* (*ncu09434*); *sad-1* (*ncu02178*); *sad-2* (*ncu04294*); *sad-3* (*ncu09211*); *sad-4* (*ncu01591*); *sad-5* (*ncu06147*); *sad-6* (*ncu06190*); *sad-7* (*ncu01917*); and *actin* (*ncu04173*).

At least 28 morphologically-distinct cell types exist in *N. crassa* (Bistis *et al.* 2003), some of which are restricted to specific stages of the life cycle. The expression pattern of *sad-7* may help infer the cell types in which it is most active. Thus, we obtained *N. crassa* RNA sequencing datasets from four independent studies (Ellison *et al.* 2011; Samarajeewa *et al.* 2014; Wang *et al.* 2014; Wu *et al.* 2014) to compare *sad-7*’s expression pattern with those of known MSUD genes. We first analyzed three RNA sequencing datasets produced from a study of wild isolates (Ellison *et al.* 2011). In this study, the wild isolates were transferred in hyphal plugs of actively growing mycelium to the center of a sheet of cellophane over Bird’s medium and cultured under constant light for 24 hr (Ellison *et al.* 2011). The *sad-7*^+^ expression levels were close to 0 RPKM in all three strains under these conditions (Figure 4, columns 1–3). This is similar for all other MSUD genes except *dcl-1*, *qip*, and *sad-6* (Figure 4, columns 1–3), the former two of which have been shown to have roles in vegetative processes (Catalanotto *et al.* 2004; Maiti *et al.* 2007). We next analyzed datasets from a study on the effect of light on liquid shaking cultures of *N. crassa* (Wu *et al.* 2014). In this study, a standard laboratory strain was cultured in the dark at 25° and 150 RPM in liquid Bird’s medium for 24 hr and then exposed to cool white fluorescent light for durations of up to 4 hr. Interestingly, expression levels were near baseline for every time point in these datasets for all MSUD genes except *dcl-1* and *sad-6* (Figure 4, columns 4–13). We next examined datasets from a study on gene expression changes during sexual development (Wang *et al.* 2014). For these datasets, a standard laboratory strain was allowed to develop protoperithecia on cellophane over carrot agar medium at 26° under constant light. On day seven, protoperithecia were fertilized with a standard laboratory strain of the opposite mating type and perithecia were allowed to develop. RNA was sequenced from protoperithecia at “time point 0” and from perithecia at seven different time points after fertilization. The relative expression levels of *qip*, *sad-4*, *sad-6*, and *sad-7* were elevated in protoperithecia with respect to the other MSUD genes in the analysis (Figure 4, column 14), and expression levels of all MSUD genes increased as sexual development progressed (Figure 4, columns 14–21). The last study included in our analysis examined RNA transcripts from crosses between *rid*^+^ or *rid*^−^ (Freitag *et al.* 2002) laboratory strains at 144 hr postfertilization (Samarajeewa *et al.* 2014). Unlike the Wang *et al.* (2014) study, which also examined the 144 hr time point, Samarajeewa *et al.* (2014) performed crosses on miracloth over synthetic crossing medium at room temperature and ambient light conditions. Despite these changes, expression levels of MSUD genes were less than twofold different across all 144 hr datasets from both studies (Figure 4, columns 21–23).

Overall, the above analysis of MSUD gene expression patterns indicates that *sad-7*’s expression pattern is most similar to that of *sad-4*. For example, both *sad-7* and *sad-4* are expressed poorly under early vegetative conditions (Figure 4, columns 1–13), upregulated in protoperithecial cultures (Figure 4, column 14), and reach maximum expression levels after fertilization (Figure 4, columns 15–23).

### SAD-7 homologs are present in a wide range of ascomycete fungi

A search of NCBI’s nonredundant protein database with the predicted sequence of *N. crassa* SAD-7 found SAD-7 homologs in many classes of ascomycete fungi. A synteny analysis suggests that many of these homologs are orthologous (related by speciation). For example, homologs of genes flanking *N. crassa sad-7* were found flanking genes of putative *sad-7* homologs in Sordariomycetes (14 of 14 species analyzed), Leotiomycetes (one of one species analyzed), Dothidiomycetes (one of one species analyzed), and Eurotiomycetes (one of one species analyzed) (Figure S1 in File S1).

**Figure 5:**
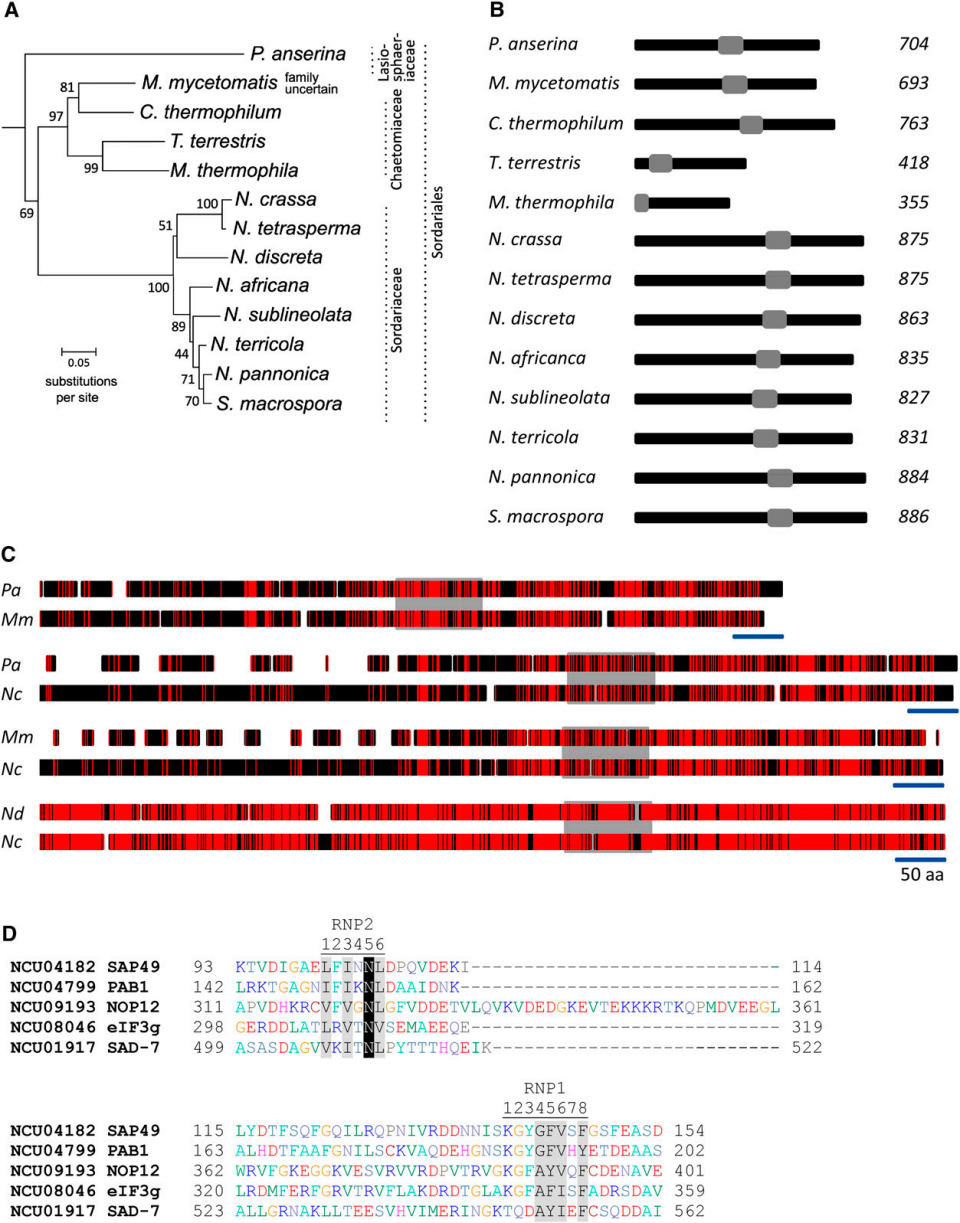
SAD-7 is a widely conserved RRM protein in ascomycete fungi. (A) A tree diagram depicting relationships between SAD-7 homologs in a clade of Sordariales. The diagram contains a subset of homologs from a more complete phylogenetic analysis presented in Figure S1 in File S1. The families of 12 of the 13 taxa in Sordariaceae, Chatomiaceae, and Lasiosphaeriaceae families are indicated (Federhen 2003). (B) RRM (NCBI CDD: cl17169) locations in SAD-7 homologs. RRM domains are indicated with gray boxes. The predicted number of amino acids in each protein is listed along the right side of the panel. (C) Graphical depictions of Clustal W (Thompson *et al.* 1994) alignments between pairs of SAD-7 homologs. Identical amino acids are indicated with red shading. Different amino acids are indicated with black shading. Gap positions are indicated with gaps. The locations of the RRM domains are indicated with a gray box. The blue scale bar is equivalent to 50 amino acids. In summary, these results show that SAD-7 is an RRM domain-containing protein conserved across a wide range of ascomycete fungi. They also suggest that the N-terminal halves of the protein have changed more than the C-terminal halves during evolution of Sordariaceae, Chatomiaceae, and Lasiosphaeriaceae fungi. (D) A manual alignment of RRM domains from five *N. crassa* proteins is shown. The residues are shaded according to the PAM120 similarity matrix. The positions of ribonucleoprotein domain 1 (RNP1) and RNP2 are indicated. Sequences can be obtained from GenBank or FungiDB with the following accession numbers: *P. anserina* (*Pa*) CAP60824.1; *M. mycetomatis* (*Mm*) KXX77199.1; *Chaetomium thermophilum* EGS22685.1; *Thielavia terrestris* AEO64981.1; *Myceliophthora thermophila* AEO61061.1; *N. crassa* (*Nc*) EAA36312.1; *N. tetrasperma* EGO51840.1; *N. discreta* (*Nd*) NEUDI 136685; *N. africana* GCA 000604205.2; *N. sublineolata* GCA 000604185.2; *N. terricola* GCA 000604245.2; *N. pannonica* GCA 000604225.2; and *Sordaria macrospora* XP 003349025.1. NCBI, National Center for Biotechnology Information; RRM, RNA recognition motif.

To gain knowledge on relationships between SAD-7 homologs in ascomycete fungi, we performed a phylogenetic analysis. A single clade representing 13 SAD-7 homologs in the Sordariales order of fungi is shown in Figure 5A. The clade contains three subclades that are consistent with current designations of the taxa into three families: the Sordariaceae, the Chaetomiaceae, and the Lasiosphaeriaceae (Federhen 2003). It should be noted that two of the four SAD-7 homologs in the Chaetomiaceae are unusually short (< 419 amino acids) (Figure 5B). We are unclear whether this is a biologically meaningful finding or a result of errors in the available genome sequences and/or annotation for these two fungi. A search of NCBI’s conserved domain database (Marchler-Bauer *et al.* 2010) identified an RRM in all 13 SAD-7 homologs. The RRM motif is found in the C-terminal half of each protein (when ignoring the two unusually short Chaetomiaceae proteins). The SAD-7 homologs in the Sordariaceae are longer than those from the other two families. For example, the shortest SAD-7 homolog in the Sordariaceae has 827 amino acids, while the longest one in the Chaetomiaceae and the Lasiosphaeriaceae has 763 amino acids. Pairwise alignments were made to identify a reason for this family-specific length difference. Alignments between *Podospora anserina* and *Madurella mycetomatis* SAD-7 revealed a high level of identity along the C-terminal half of the proteins and a comparatively low level of identity along the N-terminal half, despite the two proteins being approximately the same length (Figure 5C, top pair). In contrast, the SAD-7 homologs in *N. discreta* and *N. crassa* have a high level of identity along their entire lengths (Figure 5C, bottom pair). Interestingly, alignments between *N. crassa* and *P. anserina* SAD-7 homologs, as well as *N. crassa* and *M. mycetomatis* SAD-7 homologs, reveal a series of gaps along their N-terminal halves. These data suggest that the N-terminal half of SAD-7 lengthened in the lineage leading to the Sordariaceae and/or experienced deletions in the lineage leading to Chaetomiaceae and Lasiosphaeriaceae fungi.

We also examined the sequence of the SAD-7 RRM domain. RRM domains are ∼100 amino acids long and contain a conserved β_1_α_1_β_2_β_3_α_2_β_4_ fold (Maris *et al.* 2005). A consensus sequence called RNP1 ([RK]-G-[FY]-[GA]-[FY]-[ILV]-X-[FY]) is typically found within β_3_, while another called RNP2 ([ILV]-[FY]-[ILV]-X-N-L) is typically found within β_1_. The aromatic residues at the second position in RNP2 and the third and fifth positions in RNP1 play critical roles in RNA binding for many RRM-containing proteins. Interestingly, the RRM-domain of SAD-7 lacks an aromatic residue at position 3 in RNP1 and at position 2 in RNP2 (Figure 5D). This is not a general feature of RRM domains in *N. crassa* because other *N. crassa* RRM-containing proteins (NCU04182, NCU04799, and NCU09193) have aromatic residues at these positions. NCU04182 is a homolog of HSH49, a spliceosomal protein (Igel *et al.* 1998); NCU04799 is a homolog of PAB1, a poly(A)-binding protein (Brune *et al.* 2005); and NCU09193 is a homolog of NOP12, a protein involved in ribosome assembly (Konikkat and Woolford 2017). However, similar to SAD-7, NCU08046 has an RRM domain that lacks an aromatic residue at position 2 in RNP2. NCU08046 is a homolog of eIF3g, a protein involved in translation (Hinnebusch 2014). The biological relevance of the amino acid differences in SAD-7’s RRM domain relative to canonical RRM domains is unclear, but the residue exchanges at these positions are likely important for SAD-7 function because they are conserved among the SAD-7 orthologs in Sordariaceae, Chaetomiaceae, and Lasiosphaeriaceae fungi (Figure S2 in File S1).

### GFP-SAD-7 fusion proteins are found in the nucleus, the perinuclear region, and cytoplasmic foci of meiotic cells

**Figure 6:**
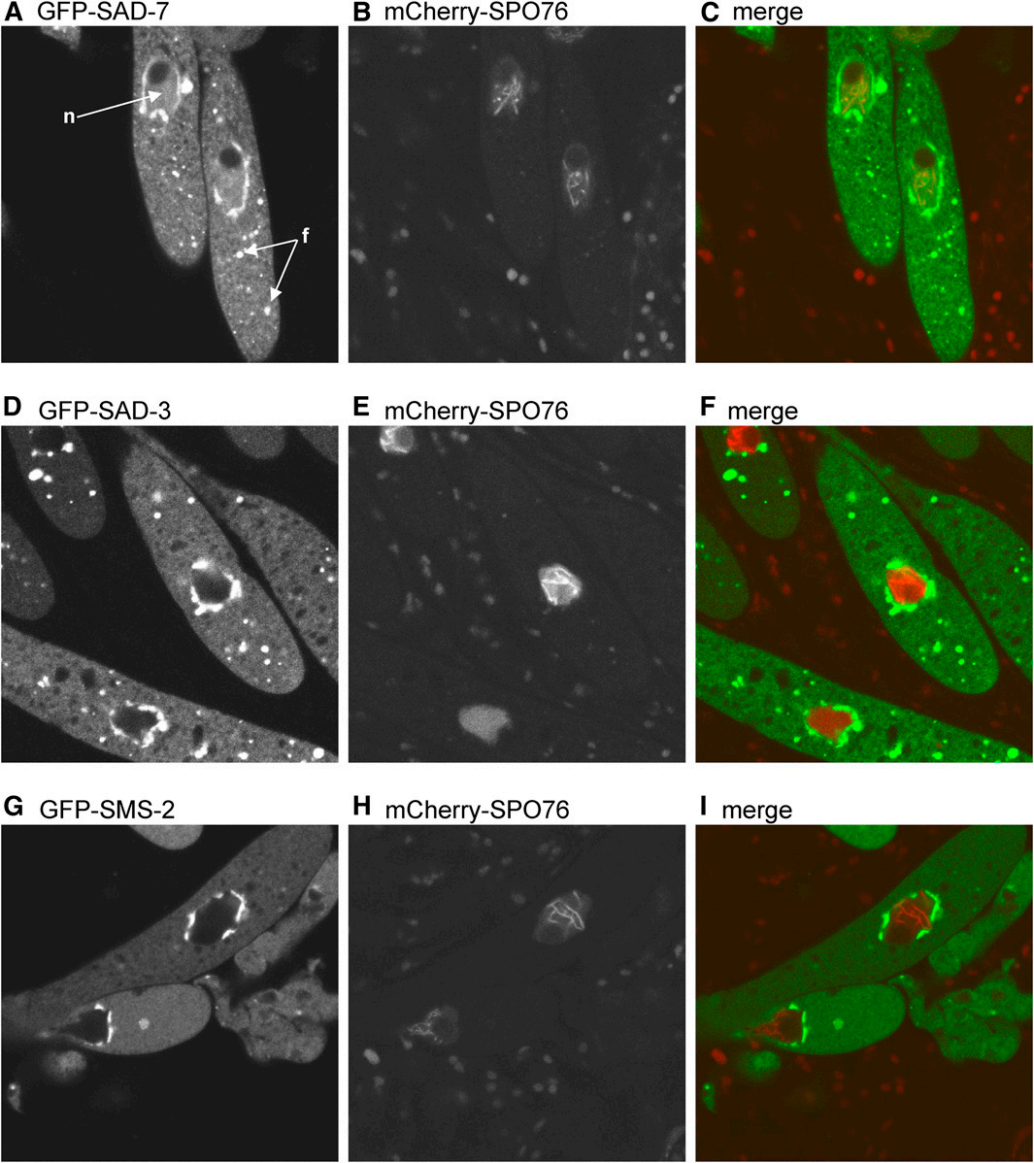
GFP-SAD-7 is detected at three different locations in the ascus. (A–C) Asci from a *gfp*-s*ad-7* × *mCherry-spo76 sad-2*^Δ^ cross are shown. When SAD-7 is tagged with GFP (GFP-SAD-7), a GFP signal is detected throughout the nucleus (*n*) except for a spherical subnuclear domain representing the nucleolus. The GFP-SAD-7 signal is most intense around the nucleus and within cytoplasmic foci (*f*). mCherry-SPO76 is used to depict the position of the chromosomes (van Heemst *et al.* 1999; Samarajeewa *et al.* 2014), while *sad-2*^Δ^ is used to allow the expression of tagged and unpaired alleles during meiosis. ISU-3334 × ISU-3817. (D–F) Asci from a *gfp*-s*ad-3* × *mCherry-spo76 sad-2*^Δ^ cross are shown. When SAD-3 is tagged with GFP (GFP-SAD-3), the GFP signal is similar to that of GFP-SAD-7 except that there is no signal within the nucleus. ISU-3329 × ISU-4261. (G–I) Asci from a *gfp*-s*ms-2* × *mCherry-spo76 sad-2*^Δ^ cross are shown. When SMS-2 is tagged with GFP, the GFP signal is strong around the nucleus but absent from within the nucleus. Cytoplasmic foci are uncommon for GFP-SMS-2. ISU-3334 × P15-22. GFP, green fluorescent protein.

The detection of unpaired DNA must occur in the nucleus. However, of the 11 previously-characterized MSUD proteins, only four have been detected within this region of the meiotic cell (SAD-5, SAD-6, CBP20, and CBP80, see *Introduction*). To examine SAD-7’s localization pattern during meiosis, we tagged its N-terminus with GFP, performed crosses, and examined the meiotic cells with confocal microscopy. For these crosses, we included an mCherry-tagged version of SPO76 (mCherry-SPO76), which localizes to meiotic chromosomes (van Heemst *et al.* 1999; Samarajeewa *et al.* 2014). In the *gfp-sad-7* × *mCherry-spo76* crosses, GFP-SAD-7 was detected at three locations: (1) within the nucleus, (2) in a ring around the nucleus, and (3) within randomly distributed cytoplasmic foci (Figure 6, A–C). To eliminate the possibility that our detection of GFP-SAD-7 within nuclei was due to an experimental artifact, we also examined the localization patterns of GFP-SAD-3 and GFP-SMS-2 (Hammond *et al.* 2011a,b). As reported in previous studies, GFP-SAD-3 and GFP-SMS-2 both formed perinuclear rings and were not detected within nuclei (Figure 6, D–I). We also noticed that GFP-SAD-7 and GFP-SAD-3 were both associated with randomly distributed cytoplasmic foci throughout the cytoplasm (Figure 6, A–F), while GFP-SMS-2 was rarely observed with such foci (Figure 6, G–I). The biological significance of these cytoplasmic foci is currently unclear.

**Figure 7:**
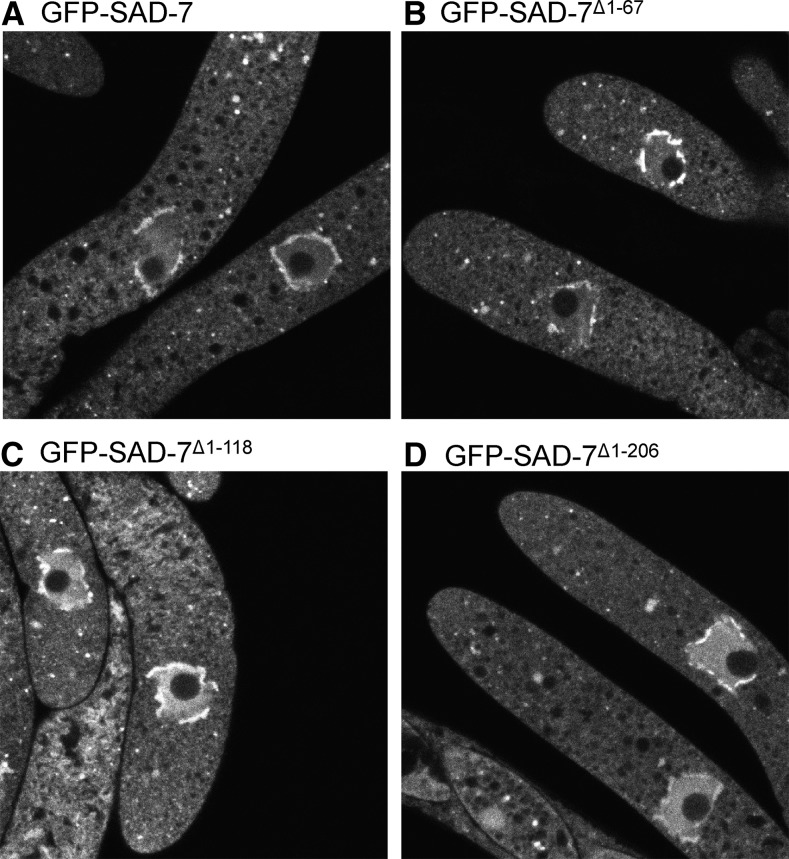
The meiotic localization pattern of GFP-SAD-7 is independent of the first 206 amino acids of the protein. A series of truncated SAD-7 proteins was created by fusing GFP to different positions from the N-terminal end of SAD-7. Amino acids prior to the fusion point were deleted in the process. Representative images of GFP signal within asci in meiotic prophase I from crosses between ISU-3334 (an *mCherry-spo76* *sad-2*^Δ^ strain) and various GFP-SAD-7 truncation strains are shown. In these crosses, GFP signal was detected within the nucleus, the perinuclear region, and cytoplasmic foci despite the loss of up to 206 amino acids from the N-terminal end of SAD-7. (A) ISU-3334 × ISU-3817, (B) ISU-3334 × ISU-4078, (C) ISU-3334 × ISU-4079, and (D) ISU-3334 × ISU-4217. GFP, green fluorescent protein.

The identification of an MSUD protein that travels between the nucleus and the cytoplasm could shed light on how nuclear events are linked to silencing processes outside of the nucleus. For example, CBP20 and CBP80 appear to be nuclear–cytoplasmic shuttling proteins involved in MSUD (Decker *et al.* 2017). If GFP-SAD-7 were also a shuttling protein, it could be possible to isolate *sad-7* mutants that are unable to travel between the nucleus and the cytoplasm. To test if N-terminal truncations of SAD-7 could disrupt its normal localization pattern, we fused GFP to positions 68 (GFP-SAD-7^Δ1–67^), 119 (GFP-SAD-7^Δ1–118^), and 207 (GFP-SAD-7^Δ1–206^) of the 875 amino acid SAD-7 protein (for each GFP tag, the amino acids prior to the fusion point were deleted). We then examined the ability of the GFP-tagged full-length protein (*i.e.*, GFP-SAD-7) and each GFP-tagged truncated protein to complete the sexual cycle when neither parent of the cross carried an untagged *sad-7*^+^ allele. While *sad-7*^Δ^ × *gfp-sad-7* crosses produced phenotypically normal perithecia and asci with ascospores (Figure 2, C and F), *sad-7*^Δ^ × *gfp-sad-7*^Δ1–67^ crosses produced beakless and barren perithecia (Figure 2D and data not shown). Beakless and barren perithecia were also produced by *sad-7*^Δ^ × *gfp-sad-7*^Δ1–118^ and the *sad-7*^Δ^ × *gfp-sad-7*^Δ1–206^ crosses (data not shown). These findings suggest that at least some of the amino acids prior to position 68 are necessary for SAD-7’s function in sexual reproduction (although we cannot discount the possibility that the GFP tag is inhibitory to the truncated SAD-7 protein but not the full-length protein). Surprisingly, despite the inability of the truncated proteins to complement the barren phenotype, all three truncated proteins displayed a meiotic localization pattern that was indistinguishable from that of full-length SAD-7 (Figure 7 and Figure S3 in File S1), suggesting that some of the amino acids prior to position 68 are required for SAD-7’s function in sexual development but none are required for its proper localization.

## Discussion

In this report, we present evidence demonstrating that *N. crassa* SAD-7 (NCU01917) is an MSUD protein. The strongest support for this hypothesis is seen in heterozygous crosses between *sad-7*^+^ and *sad-7*^Δ^, which are deficient in MSUD. This deficiency phenotype could be due to haploinsufficiency, where one copy of *sad-7*^+^ does not supply enough SAD-7 protein to the meiotic cell, and/or it could be due to a process called “silencing the silencer,” where the unpairing of *sad-7*^+^ turns the MSUD machinery against itself (*i.e.*, *sad-7*^+^) (Shiu *et al.* 2001). In either case, decreased levels of SAD-7 likely cause MSUD deficiency because it is a silencing protein.

Like many MSUD proteins, SAD-7 is required for sexual reproduction. DCL-1, QIP, SAD-1, SAD-2, SAD-3, and SMS-2 are other examples (Shiu *et al.* 2001, 2006; Lee *et al.* 2003, 2010; Alexander *et al.* 2008; Xiao *et al.* 2010; Hammond *et al.* 2011a). When an MSUD protein is required for sexual reproduction, it is not possible to determine if the protein is required (critical) for MSUD or if it simply improves its efficiency (nonessential). To understand this distinction, it is useful to consider MSUD proteins that are not required for sexual reproduction, such as SAD-5 and SAD-6. MSUD is partially suppressed in *sad-5*^+^ × *sad-5*^Δ^ crosses but completely absent in *sad-5*^Δ^ × *sad-5*^Δ^ crosses (Hammond *et al.* 2013b); therefore, SAD-5 is a critical MSUD protein. In contrast, MSUD is only partially suppressed in both *sad-6*^+^ × *sad-6*^Δ^ and *sad-6*^Δ^ × *sad-6*^Δ^ crosses (Samarajeewa *et al.* 2014); therefore, SAD-6 improves the efficiency of MSUD but is not strictly required for the process. The reason why SAD-6 is nonessential for MSUD is unknown, but it could be that its role in MSUD is shared with another protein. With respect to SAD-7, it is not possible to determine if it is more like SAD-5 (critical) or more like SAD-6 (nonessential) because sexual development stalls before meiosis when a cross is completely devoid of SAD-7.

The *sad-7*^Δ^ allele suppresses silencing of unpaired *asm-1*^+^ as strongly as the *sad-2*^Δ^ allele does; however, it suppresses silencing of unpaired *r*^+^ less well than the *sad-2*^Δ^ allele (Table 2). This finding is consistent with previous research on alleles that partially suppress silencing of unpaired *asm-1*^+^ and/or *r*^+^ in heterozygous crosses. For example, *sad-4*^Δ^, *sad-5*^Δ^, and *sad-6*^Δ^ alleles are all stronger suppressors of unpaired *asm-1*^+^ silencing than they are of unpaired *r*^+^ silencing (Hammond *et al.* 2013b; Samarajeewa *et al.* 2014). The *Neurospora* *Spore killers Sk-2* and *Sk-3*, which are MSUD suppressors, also suppress unpaired a*sm-1*^+^ silencing better than unpaired *r*^+^ silencing (Raju *et al.* 2007). These differences could be related to the level of effort that MSUD must exert to silence *asm-1*^+^ and *r*^+^ alleles. For example, perhaps *asm-1*^+^ expression is greater than that of *r*^+^ in meiotic cells. In such a scenario, MSUD may need to work harder to silence unpaired *asm-1*^+^.

SAD-7 appears to have at least one role in addition to its function in MSUD. This role should occur at or before the initiation of meiosis because sexual reproduction stalls before the appearance of elongated meiotic cells in *sad-7*^Δ^ × *sad-7*^Δ^ crosses. The fact that SAD-7 has been conserved across a diverse range of ascomycete fungi (Figure S1 in File S1) and MSUD has only been described in three species, two from *Neurospora* and one from *Fusarium* (Shiu *et al.* 2001; Ramakrishnan *et al.* 2011b; Son *et al.* 2011), suggests that SAD-7’s non-MSUD role in sexual reproduction is more broadly conserved than its role in MSUD. However, MSUD may be more common in ascomycete fungi than it is currently suggested to be by the available literature. For example, research on wild *N. crassa* isolates has shown that MSUD can be difficult to detect, even when it is known to exist within a species (Ramakrishnan *et al.* 2011a). Therefore, it is possible that SAD-7 performs similar functions in MSUD and sexual reproduction in a diverse range of ascomycete fungi.

By comparing SAD-7 orthologs from three families in the Sordariales class of ascomycete fungi, we found that while the N-terminal halves of SAD-7 proteins have undergone the most diversification, the C-terminal halves have changed comparatively little. The simplest explanation for this is that the N-terminal halves mediate interactions with lineage-specific proteins, while the C-terminal halves perform a similar function among the various lineages. Accordingly, the RRM domain of each SAD-7 is found in the C-terminal half of each protein (ignoring the two unusually short Chaetomiaceae SAD-7s). A sequence level analysis of the RRM domain in 12 SAD-7 orthologs revealed all to be missing two aromatic residues typical of canonical RRM domains. The current reason for this is unclear, but similar variations have been identified in other RRM sequences. These variations appear to be due to the diverse functions of RRM domains, which include RNA binding, protein binding, and/or directing cellular localization (Cléry *et al.* 2008; Cassola *et al.* 2010; Muto and Yokoyama 2012). Future investigation of the binding affinities of SAD-7’s RRM domain could help us understand SAD-7’s specific role in MSUD and sexual reproduction.

Perhaps the most intriguing finding concerning SAD-7 thus far is its peculiar localization pattern relative to other MSUD proteins. Typically, MSUD proteins are localized by N-terminal or C-terminal tagging of the protein with GFP (Lee *et al.* 2010; Hammond *et al.* 2011a,b, 2013b; Samarajeewa *et al.* 2014). This has generally produced localization patterns that are nuclear or extranuclear, but not both. We propose that GFP-SAD-7’s nuclear and extranuclear localizations are related to its role in MSUD and/or sexual reproduction. Interestingly, SAD-7 function appears to require the 67 amino acids at its N-terminal end because fusing GFP to the 68th amino acid (while eliminating the previous 67) abolishes spore production. This finding was made during an attempt to alter the localization pattern of GFP-SAD-7. For example, if the loss of the first 67 amino acids eliminated our ability to detect SAD-7 in either the nucleus or the cytoplasm, it would have added credence to the hypothesis that SAD-7 is a shuttling protein. Surprisingly, all three of the truncated SAD-7 proteins examined in this study displayed a localization pattern similar to the full-length protein, despite none being sufficient for sexual reproduction. One possibility is that SAD-7 localization is determined by residues at and after position 207, the site of the GFP fusion in GFP-SAD-7^∆1–206^. However, because our analysis of the GFP-SAD-7 truncation proteins was performed in the presence of the full-length SAD-7 (*e.g.*, *sad-7^+^* × *gfp-sad-7*^∆1–67^), one possibility is that SAD-7 forms a homodimer through interactions between residues after position 206. In this scenario, localization signals from the full-length SAD-7 would direct SAD-7/GFP-SAD-7^∆1–67^, SAD-7/GFP-SAD-7^∆1–118^, and SAD-7/GFP-SAD-7^∆1–206^ dimers to the proper location in the cell.

The presence of SAD-7 in nuclear and extranuclear regions of meiotic cells provides a clue toward understanding how the nuclear aspects of MSUD are linked to perinuclear and cytoplasmic aspects of the process. The MSUD model suggests that aRNAs are transcribed from unpaired DNA and delivered to silencing proteins present in a perinuclear ring around the nucleus. Given SAD-7’s nuclear and extranuclear localization pattern, it is conceivable that SAD-7 participates in the latter process. Future studies on the binding affinities of SAD-7’s RRM domain and the identification of proteins that interact with SAD-7 could help determine if and how SAD-7 links nuclear and extranuclear aspects of MSUD.

## Supplementary Material

Supplemental material is available online at www.g3journal.org/lookup/suppl/doi:10.1534/g3.117.041848/-/DC1.
