# Supplementary material for: An RNA Recognition Motif-Containing Protein Functions in Meiotic Silencing by Unpaired DNA
Source: G3 (Bethesda). 2017 Jun 30;7(8):2871–82. doi: 10.1534/g3.117.041848 (PMC5555490; doi:10.1534/g3.117.041848)
Supplement: Supplementary file 1 [file 2871File001.pdf]

Table S1 Oligonucleotides used in this study

Name	Sequence (5′ to 3′)
Primers for constructing gfp-sad-7	
SAD-7-E	CATTTGCTCTTGCCCCTCTGCTT
SAD-7-NGFP1	GCAGCCTGAATGGCGAATGGACGCGCAGGATGGTTGGTTAGCGGTCAGG
SAD-7-NGFP3	CAGGAGCGGGTGCGGGTGCTGGAGCGATGGCGGACATCAAACGCA
SAD-7-I	TTAGGCACGAAGCCCTGACCATT
SAD-7-J	AGTTGTTCTGACGGTTGGCTGCT
SAD-7-K	TTGTTGAAAAGATTGCGTTGCTTGAGG
Primers for constructing gfp-sad-7$^{\Delta 1\text{-}67}$	
SAD-7-E	CATTTGCTCTTGCCCCTCTGCTT
SAD-7-NGFP1	GCAGCCTGAATGGCGAATGGACGCGCAGGATGGTTGGTTAGCGGTCAGG
SAD-7-L	CAGGAGCGGGTGCGGGTGCTGGAGCGCCGAATGCTGTCATGGCTCAAGAT
SAD-7-M	TCAGCAATACGAGAAGGACGGTTT
SAD-7-J	AGTTGTTCTGACGGTTGGCTGCT
SAD-7-O	TAGGCACGAAGCCCTGACCATT
Primers for constructing gfp-sad-7$^{\Delta 1\text{-}118}$	
SAD-7-E	CATTTGCTCTTGCCCCTCTGCTT
SAD-7-NGFP1	GCAGCCTGAATGGCGAATGGACGCGCAGGATGGTTGGTTAGCGGTCAGG
SAD-7-P	CAGGAGCGGGTGCGGGTGCTGGAGCGGGAAACCACGGACGCGAGCAGAA
SAD-7-M	TCAGCAATACGAGAAGGACGGTTT
SAD-7-J	AGTTGTTCTGACGGTTGGCTGCT
SAD-7-Q	GATTGCGCGAAGGCTAGAGGAC
Primers for constructing gfp-sad-7$^{\Delta 1\text{-}206}$	
SAD-7-E	CATTTGCTCTTGCCCCTCTGCTT
SAD-7-NGFP1	GCAGCCTGAATGGCGAATGGACGCGCAGGATGGTTGGTTAGCGGTCAGG
SAD-7-R	CAGGAGCGGGTGCGGGTGCTGGAGCGAATCAGCAGGCAGGCATCATTTAC
SAD-7-M	TCAGCAATACGAGAAGGACGGTTT
SAD-7-J	AGTTGTTCTGACGGTTGGCTGCT
SAD-7-S	GGCGGTGGTTGAGAAGGAAGTG

The above oligonucleotides were used as primers for constructing four *gfp-sad-7* tagging vectors with DJ-PCR as described by Hammond *et al.* (2011). For each set of six primers listed above, the first two were used to amplify the left flank, the middle two were used to amplify the right flank, and the last two were used as nested primers to amplify the final vector.

Figure S1 SAD-7 homologs are present in a wide range of ascomycete fungi. The sequence of *N. crassa* SAD-7 was used as the query in a blastp search (Altschul *et al.* 1997) of NCBI's non-redundant protein database. The highest scoring subject sequence was selected for each *Neurospora* species present in the results (*N. crassa* and *N. tetrasperma*). The single most significant subject sequence for each other genus represented in the results was also selected. Each selected sequence was then used as the query in a reciprocal blastp search of all predicted *N. crassa* proteins. Those that identified SAD-7 as the most significant match were included in the SAD-7 phylogenetic analysis. SAD-7 sequences for *N. discreta*, *N. africana*, *N. sublineolata*, *N. terricola*, and *N. pannonica* were also included. These sequences were obtained from fungiDB (*N. discreta*) or draft genome assemblies downloaded from NCBI. Sequences were imported into MEGA (7.0.18) (Kumar *et al.* 2016) and aligned with MUSCLE using default settings. Positions having less than 95% coverage were eliminated, and a Neighbor-Joining tree was constructed from the 220 remaining positions in MEGA using the Poisson correction method (Zuckerland and Pauling 1965). A bootstrap test (Felsenstein 1985) with 1000 replicates was performed. Each tip of the tree was labeled with a GenBank accession number, FungiDB number, or Genome Assembly number (NCBI) for the corresponding sequence. The species name and the Expect value for each sequence, which was obtained from the original blastp search of NCBI's non-redundant protein database with SAD-7 as the query, were also included in the labels. NCBI's taxonomy database (Federhen 2003) was used to organize clades by phylum as well as by class for sequences from ascomycete fungi. Syntenic relationships between some of the putative *sad-7* homologs were examined. Fourteen genes, including *N. crassa's* version, were confirmed to be adjacent to a gene encoding an ARP2/3 complex protein (red stars). One *sad-7* homolog (blue star) was four genes away from a ubiquitin C-terminal

hydrolase gene, while *N. crassa sad-7* is two genes away from a similar gene. Putative homologs of the four genes immediately surrounding *sad-7* in the *N. crassa* genome (*ncu01915*, *ncu01916*, *ncu01918*, and *ncu01919*) were not found near the putative *sad-7* homolog from *M. circinelloides* (green circle), suggesting the relationship between these two putative genes may not be biologically significant. Syntenic relationships were not investigated for other sequences in the tree.

Figure S2

```
                              RNP2                                          RNP1
                            123456                                        12345678
Nc   SAD-7   496 CSQASASDAGVVKITNLPYTTTHQEIKALLGRNAKLLT--EESVHVIMERINGKTQDAYIEFCSQDD 560
Nt   SAD-7   496 CSQASASDAGVVKITNLPYTTTHQEIKALLGRNAKLLT--EESVHVIMERINGKTQDAYVEFCSQDD 560
Nd   SAD-7   486 CSQASASNAGVVKIRNIPYTTTHQEIKAFLGRTAKILPDIDEPLHVIMERINGKTQDAYIEFCSQDD 552
Na   SAD-7   460 CSQASASDAGVVKIKNIPYMTTHQEIKAFLGRNSKIQNDSQEPIHVIMERISGKTQEAYVEFFSQDD 526
Ns   SAD-7   447 CSQASASDFGVIKIKNIPYTTTHQEIKAFLGRNSKLLNDAQEPVHVIMERISGKTQEAYVEFFHQED 511
Nte  SAD-7   451 CSQASASDAGVVKIKNIPYMTTHQEIKAFLGRNSKILNDTQEPVHVIMERISGKTQEAYVEFFSQDD 517
Np   SAD-7   504 CSQASASDAGVVKIKNIPYMTTHQEIKAFLGRNSKILNDAQEPVHVIMERISGKTQEAYVEFFSQDD 570
Sm   SAD-7   506 CSQATASDAGVVKIKNIPYMTTHQEIKAFLGRNSKILNDPQEPVHVIMERISGKTQEAYVEFFSQDD 572
Pa   SAD-7   317 SSQSMPAVWGVIKISNIPFGTMRAEVIAMLGRNSKIITDAQEGVHIIMERVTSKTGDAFVEFSSIHA 383
Ct   SAD-7   399 CTQATPVPYGVVKICNIPFGTKRSEIIAFLGRNSKILNDNQEPVHIIMERVTSKTQDAYVEFMTLHD 465
Mm   SAD-7   329 CSQAGPVSHGVVKIRNIPFGTKRAEIIAFLGRNSKILNDNQEPVHIIMERVTSKTHDAYVEFMTLPD 395
Tt   SAD-7    46 TTQGGPVSHGVIKIKNIPFATKRAEIIAFLGRNSRILNDNQEPVHIIMERVSSKTQDCYVEFITPQD 112
```

Alignment of RRM domains in a clade of Sordariales fungi. Amino acids are shaded according to the BLOSUM62 similarity matrix. Positions of the RNP1 and RNP2 motifs are indicated. Sequences can be obtained from GenBank or FungiDB with the following accession numbers: *Neurospora crassa* (*Nc*) EAA36312.1; *Neurospora tetrasperma* EGO51840.1; *Neurospora discreta* (*Nd*) NEUDI 136685; *Neurospora africana* (*Na*) GCA 000604205.2; *Neurospora sublineolata* (*Ns*) GCA 000604185.2; *Neurospora terricola* (*Nte*) GCA 000604245.2; *Neurospora pannonica* (*Np*) GCA 000604225.2; *Sordaria macrospora* (*Sm*) XP 003349025.1; *Podospora anserina* (*Pa*) CAP60824.1; *Chaetomium thermophilum* (*Ct*) EGS22685.1; *Madurella mycetomatis* (*Mm*) KXX77199.1; and *Thielavia terrestris* (*Tt*) AEO64981.1.

Figure S1

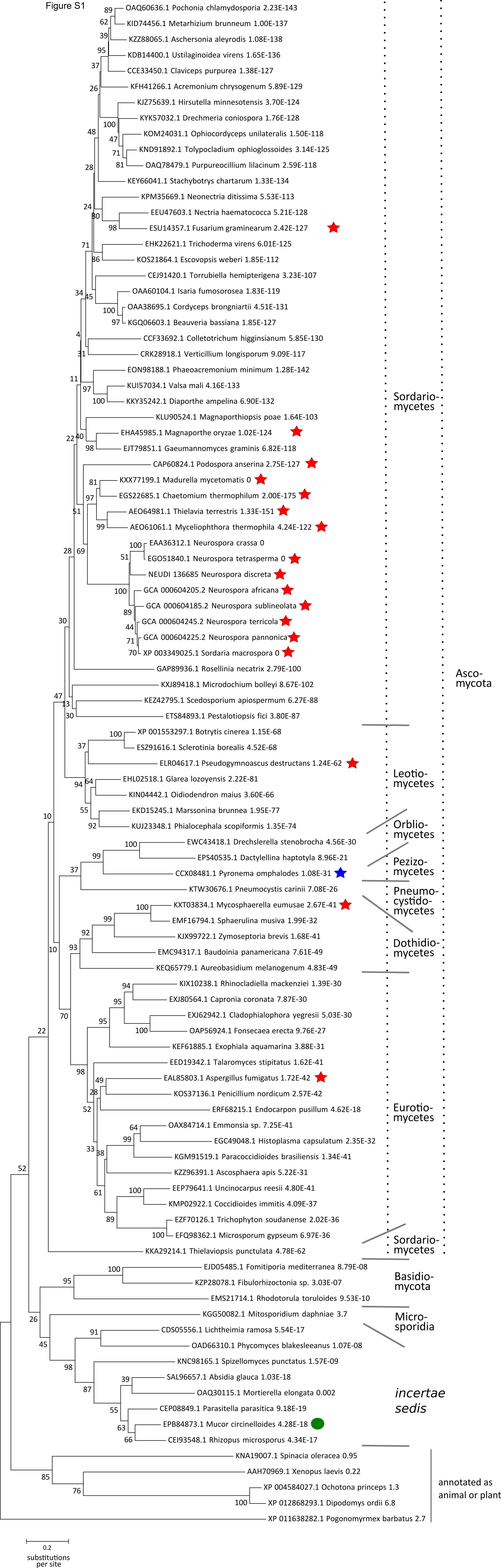
OAQ60636.1 Pochonia chlamydosporia 2.23E-143
KID74456.1 Metarhizium brunneum 1.00E-137
KZZ88065.1 Aschersonia aleyrodis 1.08E-138
KDB14400.1 Ustilaginoidea virens 1.65E-136
CCE33450.1 Claviceps purpurea 1.38E-127
KFH41266.1 Acremonium chrysogenum 5.89E-129
KJZ75639.1 Hirsutella minnesotensis 3.70E-124
KYK57032.1 Drechmeria coniospora 1.76E-128
KOM24031.1 Ophiocordyceps unilateralis 1.50E-118
KND91892.1 Tolypocladium ophioglossoides 3.14E-125
OAQ78479.1 Purpureocillium lilacinum 2.59E-118
KEY66041.1 Stachybotrys chartarum 1.33E-134
KPM35669.1 Neonectria ditissima 5.53E-113
EEU47603.1 Nectria haematococca 5.21E-128
ESU14357.1 Fusarium graminearum 2.42E-127
EHK22621.1 Trichoderma virens 6.01E-125
KOS21864.1 Escovopsis weberi 1.85E-112
CEJ91420.1 Torrubiella hemipterigena 3.23E-107
OAA60104.1 Isaria fumosorosea 1.83E-119
OAA38695.1 Cordyceps brongniartii 4.51E-131
KGQ06603.1 Beauveria bassiana 1.85E-127
CCF33692.1 Colletotrichum higginsianum 5.85E-130
CRK28918.1 Verticillium longisporum 9.09E-117
EON98188.1 Phaeoacremonium minimum 1.28E-142
KUI57034.1 Valsa mali 4.16E-133
KKY35242.1 Diaporthe ampelina 6.90E-132
KLU90524.1 Magnaporthiopsis poae 1.64E-103
EHA45985.1 Magnaporthe oryzae 1.02E-124
EJT79851.1 Gaeumannomyces graminis 6.82E-118
CAP60824.1 Podospora anserina 2.75E-127
KXX77199.1 Madurella mycetomatis 0
EGS22685.1 Chaetomium thermophilum 2.00E-175
AEO64981.1 Thielavia terrestris 1.33E-151
AEO61061.1 Myceliophthora thermophila 4.24E-122
EAA36312.1 Neurospora crassa 0
EGO51840.1 Neurospora tetrasperma 0
NEUDI 136685 Neurospora discreta
GCA 000604205.2 Neurospora africana
GCA 000604185.2 Neurospora sublineolata
GCA 000604245.2 Neurospora terricola
GCA 000604225.2 Neurospora pannonica
XP 003349025.1 Sordaria macrospora 0
GAP89936.1 Rosellinia necatrix 2.79E-100
KXJ89418.1 Microdochium bolleyi 8.67E-102
KEZ42795.1 Scedosporium apiospermum 6.27E-88
ETS84893.1 Pestalotiopsis fici 3.80E-87
XP 001553297.1 Botrytis cinerea 1.15E-68
ESZ91616.1 Sclerotinia borealis 4.52E-68
ELR04617.1 Pseudogymnoascus destructans 1.24E-62
EHL02518.1 Glarea lozoyensis 2.22E-81
KIN04442.1 Oidiodendron maius 3.60E-66
EKD15245.1 Marssonina brunnea 1.95E-77
KUJ23348.1 Phialocephala scopiformis 1.35E-74
EWC43418.1 Drechslerella stenobrocha 4.56E-30
EPS40535.1 Dactylellina haptotyla 8.96E-21
CCX08481.1 Pyronema omphalodes 1.08E-31
KTW30676.1 Pneumocystis carinii 7.08E-26
KXT03834.1 Mycosphaerella eumusae 2.67E-41
EMF16794.1 Sphaerulina musiva 1.99E-32
KJX99722.1 Zymoseptoria brevis 1.68E-41
EMC94317.1 Baudoinia panamericana 7.61E-49
KEQ65779.1 Aureobasidium melanogenum 4.83E-49
KIX10238.1 Rhinocladiella mackenziei 1.39E-30
EXJ80564.1 Capronia coronata 7.87E-30
EXJ62942.1 Cladophialophora yegresii 5.03E-30
OAP56924.1 Fonsecaea erecta 9.76E-27
KEF61885.1 Exophiala aquamarina 3.88E-31
EED19342.1 Talaromyces stipitatus 1.62E-41
EAL85803.1 Aspergillus fumigatus 1.72E-42
KOS37136.1 Penicillium nordicum 2.57E-42
ERF68215.1 Endocarpon pusillum 4.62E-18
OAX84714.1 Emmonsia sp. 7.25E-41
EGC49048.1 Histoplasma capsulatum 2.35E-32
KGM91519.1 Paracoccidioides brasiliensis 1.34E-41
KZZ96391.1 Ascosphaera apis 5.22E-31
EEP79641.1 Uncinocarpus reesii 4.80E-41
KMP02922.1 Coccidioides immitis 4.09E-37
EZF70126.1 Trichophyton soudanense 2.02E-36
EFQ98362.1 Microsporum gypseum 6.97E-36
KKA29214.1 Thielaviopsis punctulata 4.78E-62
EJD05485.1 Fomitiporia mediterranea 8.79E-08
KZP28078.1 Fibulorhizoctonia sp. 3.03E-07
EMS21714.1 Rhodotorula toruloides 9.53E-10
KGG50082.1 Mitosporidium daphniae 3.7
CDS05556.1 Lichtheimia ramosa 5.54E-17
OAD66310.1 Phycomyces blakesleeanus 1.07E-08
KNC98165.1 Spizellomyces punctatus 1.57E-09
SAL96657.1 Absidia glauca 1.03E-18
OAQ30115.1 Mortierella elongata 0.002
CEP08849.1 Parasitella parasitica 9.18E-19
EPB84873.1 Mucor circinelloides 4.28E-18
CEI93548.1 Rhizopus microsporus 4.34E-17
KNA19007.1 Spinacia oleracea 0.95
AAH70969.1 Xenopus laevis 0.22
XP 004584027.1 Ochotona princeps 1.3
XP 012868293.1 Dipodomys ordii 6.8
XP 011638282.1 Pogonomyrmex barbatus 2.7
Sordario-mycetes
Asco-mycota
Leotio-mycetes
Orbilio-mycetes
Pezizo-mycetes
Pneumo-cystido-mycetes
Dothidio-mycetes
Euroti-mycetes
Sordario-mycetes
Basidio-mycota
Micro-sporidia
incertae sedis
annotated as animal or plant
0.2
substitutions per site

Figure S3

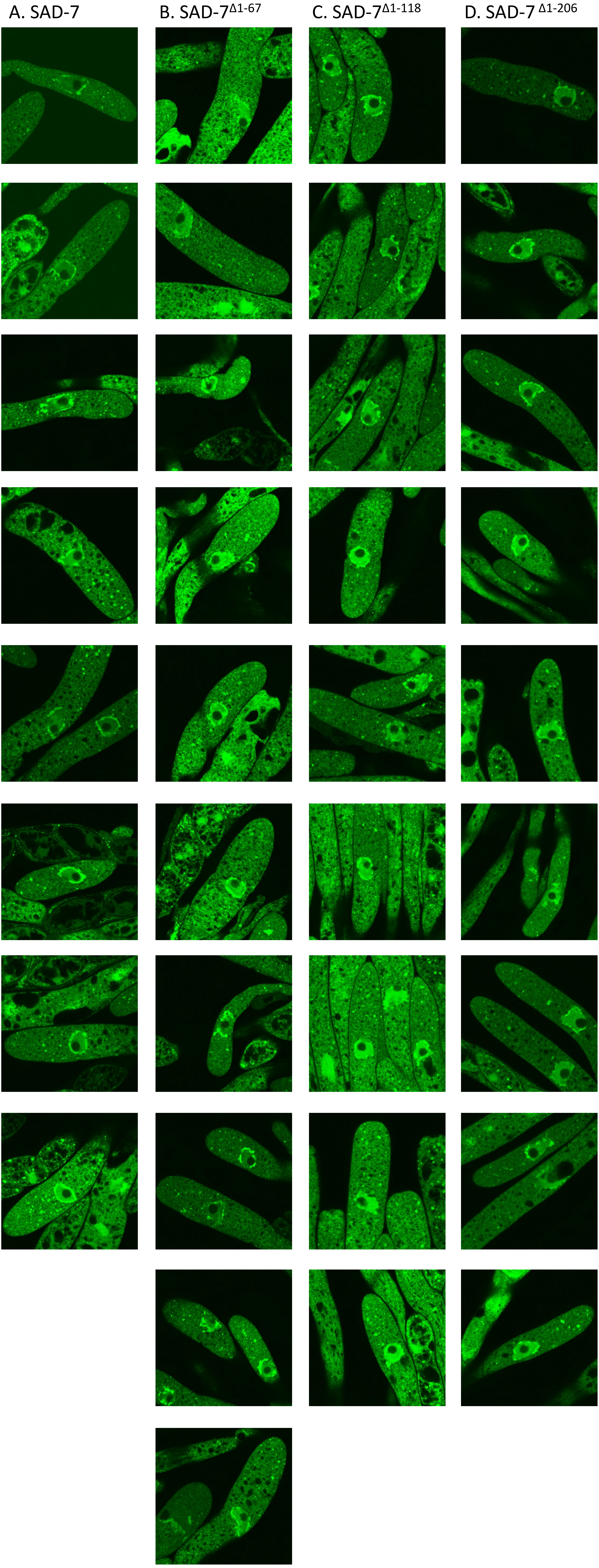

Figure S3 The meiotic localization pattern of GFP-SAD-7 does not depend on the first 206 amino acids of the protein. A series of truncated SAD-7 proteins was created by fusing GFP to different positions from the N-terminal end of SAD-7. Amino acids prior to the fusion point were deleted during the process. Representative images of the GFP signal from asci undergoing meiosis I are shown in Figure 7. Additional images for each GFP-SAD-7 are shown here. (A) GFP is fused to the first amino acid of SAD-7 (ISU-3334 × ISU-3817). (B) GFP is fused to the 68th amino acid of SAD-7 (ISU-3334 × ISU-4078). (C) GFP is fused to the 119th amino acid of SAD-7 (ISU-3334 × ISU-4079). (D) GFP is fused to the 207th amino acid of SAD-7 (ISU-3334 × HDS36.1.1). The female strain in each cross is ISU-3334. This strain carries a *sad-2*$^{\Delta}$ allele, which allows a *gfp-sad-7* transgene to be expressed despite being unpaired during meiosis.

LITERATURE CITED

Altschul, S. F., T. L. Madden, A. A. Schäffer, J. Zhang, Z. Zhang *et al.*, 1997 Gapped BLAST and PSI-BLAST: a new generation of protein database search programs. Nucleic Acids Res. 25: 3389–3402.

Federhen, S., 2003 The Taxonomy Project, in *The NCBI Handbook [Internet]*, National Center for Biotechnology Information (US).

Felsenstein, J., 1985 Confidence limits on phylogenies: An approach using the bootstrap. Evolution 39: 783–791.

Hammond, T. M., H. Xiao, E. C. Boone, T. D. Perdue, P. J. Pukkila *et al.*, 2011 SAD-3, a putative helicase required for meiotic silencing by unpaired DNA, interacts with other components of the silencing machinery. G3 1: 369–376.

Kumar, S., G. Stecher, and K. Tamura, 2016   MEGA7: Molecular evolutionary genetics analysis version 7.0 for bigger datasets. Mol. Biol. Evol. 33: 1870–1874.

Zuckerland, E., and L. Pauling, 1965 Evolutionary divergence and convergence in proteins, pp. 97–166 in *Evolving Genes and Proteins,* edited by V. Bryson and H. J. Vogel. Academic Press, New York.
